# Molecular Heterogeneity in BRAF-Mutant Gliomas: Diagnostic, Prognostic, and Therapeutic Implications

**DOI:** 10.3390/cancers15041268

**Published:** 2023-02-16

**Authors:** Benoit Lhermitte, Thibaut Wolf, Marie Pierre Chenard, Andres Coca, Julien Todeschi, François Proust, Edouard Hirsch, Roland Schott, Georges Noel, Eric Guerin, Damien Reita, Agathe Chammas, Alexandra Salmon, Sophie Martin, Monique Dontenwill, Natacha Entz-Werlé

**Affiliations:** 1 Pathology Department, University Hospital of Strasbourg, 67098 Strasbourg, France; 2 UMR CNRS 7021, Laboratory Bioimaging and Pathologies, Tumoral Signaling and Therapeutic Targets, Faculty of Pharmacy, 67405 lllkirch, France; 3 Centre de Ressources Biologiques, University Hospital of Strasbourg, 67098 Strasbourg, France; 4 Neurosurgery Department, University Hospital of Strasbourg, 67098 Strasbourg, France; 5 Neurology Department, University Hospital of Strasbourg, 67098 Strasbourg, France; 6 Oncology Department, ICANS (Institut de CANcérologie Strasbourg Europe), University of Strasbourg, 67200 Strasbourg, France; 7 Radiotherapy Department, ICANS, University of Strasbourg, 67200 Strasbourg, France; 8 Oncobiology Platform, Laboratory of Biochemistry, University Hospital of Strasbourg, 67098 Strasbourg, France; 9 Radiology Department, University Hospital of Strasbourg, 67098 Strasbourg, France; 10 Pediatric Onco-Hematology Unit, University Hospital of Strasbourg, 67098 Strasbourg, France

**Keywords:** MAPK-induced gliomas, BRAF p.V600E mutation, oncogene-induced senescence, driver, passenger molecular alterations, CDKN2A, BRAF inhibitor, MEK inhibitor

## Abstract

**Simple Summary:**

Drugs targeting activating *BRAF* mutations have transformed the prognosis and treatment of MAPK-pathway-induced cancers. In neuro-oncology, the better knowledge of the MAPK pathway’s involvement in gliomagenesis offers hope in a subset of brain cancers where conventional therapies have produced disappointing results. The temptation to use BRAF inhibitors alone or in combination in cerebral mutant tumors is high and is providing survival benefit in trials. Nonetheless, it is currently not clear what kind of gliomas can be treated and when the patient will benefit from these therapies in terms of permanent curability. This review will summarize the up-to-date literature regarding BRAF-altered gliomas, their molecular diagnosis, their prognosis, their associated molecular alterations, and how potentially treat those tumors.

**Abstract:**

Over the last few decades, deciphering the alteration of molecular pathways in brain tumors has led to impressive changes in diagnostic refinement. Among the molecular abnormalities triggering and/or driving gliomas, alterations in the MAPK pathway reign supreme in the pediatric population, as it is encountered in almost all low-grade pediatric gliomas. Activating abnormalities in the MAPK pathway are also present in both pediatric and adult high-grade gliomas. Across those alterations, *BRAF* p.V600E mutations seem to define homogeneous groups of tumors in terms of prognosis. The recent development of small molecules inhibiting this pathway retains the attention of neurooncologists on BRAF-altered tumors, as conventional therapies showed no significant effect, nor prolonged efficiency on the high-grade or low-grade unresectable forms. Nevertheless, tumoral heterogeneity and especially molecular alteration(s) associated with MAPK-pathway abnormalities are not fully understood with respect to how they might lead to the specific dismal prognosis of those gliomas and/or affect their response to targeted therapies. This review is an attempt to provide comprehensive information regarding molecular alterations related to the aggressiveness modulation in *BRAF*-mutated gliomas and the current knowledge on how to use those targeted therapies in such situations.

## 1. Introduction

**Figure 1:**
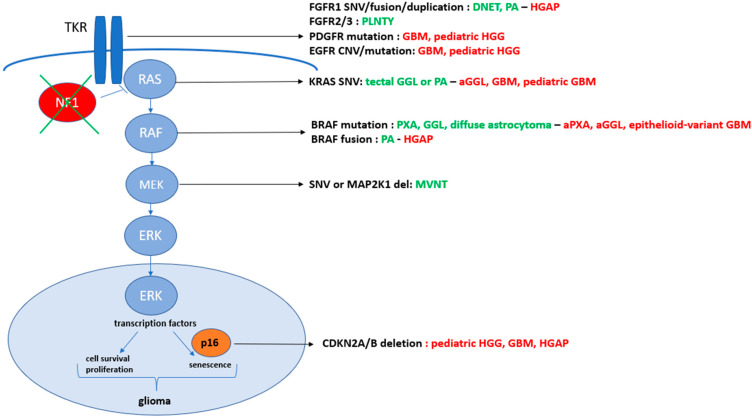
Summary of the molecular abnormalities in the MAPK pathway driving low- and high-grade gliomas. The low-grade entities are listed in green with their more frequent paired molecular aberrations, whereas the high-grade gliomas (HGGs) are described in red and LGG in greed. The alterations are listed in black color. DNET, dysembryoplastic neuroepithelial; PA, pilocytic astrocytoma; HGAP, high-grade astrocytoma with piloïd features; PLNTY, polymorphous low-grade neuroepithelial tumor of the young; GBM, glioblastoma; HGG, high-grade glioma; GGL, ganglioglioma; aGGL, anaplastic GGL; PXA, pleomorphic xanthoastrocytomas; aPXA, anaplastic PXA; MVNT, multinodular and vacuolating neuronal tumors.

In the last few decades, glioma stratification for patient diagnosis and management has impressively evolved [1]. The greater understanding of their biology has led to a new histo-molecular classification, going beyond tumor morphology, and subsequently improved accurate diagnostic procedures and targeted treatments [1,2]. In the heterogeneous group of gliomas, the deregulation of the MAPK pathway is frequently evidenced in pediatric low-grade gliomas (PLGGs), but also in rarer adult and pediatric high-grade gliomas (HGGs). Transducing the signal from the cell membrane to the nucleus, this molecular signaling encompasses proteins, whose paired genes are frequently mutated or fused in gliomas. In fact, the physiological activation of the MAPK pathway results from the ligand-dependent stimulation of tyrosine-kinase transmembrane receptors (TKR), which belong mostly to the HER (such as EGFR, FGFR or PDGFR families). The receptor homo- or heterodimerization leads then to downstream cascade phosphorylation and activation, involving RAS, RAF kinase, MEK1/2 and, finally, ERK. Activated ERK proteins translocate to the nucleus, where they phosphorylate and regulate various transcription factors, promoting changes in gene expression. This signal transduction contributes to the regulation of normal cellular processes [3], such as proliferation, differentiation, survival, or senescence [4,5]. In gliomagenesis, the MAPK pathway balances cellular pro-tumoral (an increased proliferation and a prolonged cell survival) and anti-tumoral (cell differentiation and a senescence induction) effects. The dual role of MAPK deregulation is inducing tumors that are mostly low-grade gliomas and tend to stay that way unless other genetic alterations occur [6]. MAPK pathway dysregulation is driven by gene fusions or mutations arising in all genes of the cascade, as described in Figure 1 [1]. The two main actionable therapeutic targets are MEK and BRAF activations, providing alternative therapeutic strategies in the case of unsuccessful standard chemotherapies. The recent advances in genomic and transcriptomic fields have supplied larger information about their specific abnormalities in gliomas. Nevertheless, little is known about the associated biomarkers involved potentially in gliomagenesis modifications or acceleration and therapeutic resistances along the patient journey with BRAF-altered gliomas. 

This review aims to provide comprehensive data about the role of the MAPK pathway, focusing on *BRAF* gene mutation, its involvement in the glioma tumor initiation, prognosis, progression, and treatment. We include the associated molecular pathways that are deregulated, where we will focus on their propensity to underlie resistance to the new therapeutic agents targeting MAPK upregulation.

## 2. *BRAF* Mutations

The more frequent established aberrations of the MAPK pathway encountered in gliomas are related to the RAF serine-threonine kinases. Three isoforms of RAF kinases exist and are named A-RAF, B-RAF, and C-RAF. Commonly, they bear the same general structure consisting of an N-terminal regulatory domain that physiologically inhibits the C-terminal kinase domain. This latter domain is activated when RAS binds to the RAF N-terminal end. The predominance of the BRAF-altered tumors is subsequent to B-RAF’s specific role in downstream MEK activation, unlike A-RAF and C-RAF. In addition, B-Raf protein possesses only two kinase activation sites, whereas the two other isoforms have four sites. These protein characteristics explain how it might be easier to dysregulate B-RAF with single point mutations or specific fusions than in the other two protein isoforms [4,6]. 

In low-grade gliomas (LGGs), two main types of *BRAF* alterations are described and considered as main drivers. First, a cytogenetic abnormality led to the loss of the N-terminal regulator domain of B-RAF, whereas the C-terminal kinase domain is retained, resulting in a constitutive activation of B-RAF independently from RAS activation. This molecular aberration is a tandem duplication on chromosome 7q34 involving *BRAF* and a centromeric gene, namely *KIAA1549* or, rarely, *FAM131B*. Other transcripts are more and more frequently being described [1,7]. The fused tumors are specifically and mostly pilocytic astrocytomas (PAs) [8]. The second way to activate the MAPK pathway in gliomas is a *BRAF* point mutation in its C-terminal domain, consisting generally of a substitution of a valine (V) by a glutamic acid (E) at amino acid 600. The BRAF p.V600E mutation leads to a constant phosphorylation of the threonine in position 599 and the serine in position 602. Subsequently, the B-Raf mutated protein permanently activates MEK and ERK, independently from RAS stimulation [9]. This mutation is a class I BRAF alteration. In contrast to *BRAF* duplications, *BRAFv600e* mutation is significantly associated with both low- and high-grade glial histopathologies [1,8,9,10]. 

In the LGG subtypes, pathological activation of the MAPK pathway may rarely result in mutations or gene fusions occurring in downstream effectors including ROS1, ALK, KRAS, MAP2K1 or NF1 [10], as described in Figure 1. Extremely rare fusions are described with the *RAF1* gene in PA [8].

In MAPK-activated HGGs, beyond the *BRAFv600* mutants, abnormalities can be observed in TKRs and in KRAS or with induced proliferation throughout *CDKN2A* deletion. HGGs exhibit other *BRAF* mutations extremely rarely, considered as class II and III mutations [11,12]. Mostly, A-RAF and C-RAF/RAF1 are overexpressed in HGGs, leading to a more aggressive cell phenotype and a worse patient outcome, but no mutations have so far been diagnosed for those RAF isoforms [13,14]. RAF1 was also known as a fusion partner of ATG7 [8,14,15].

## 3. Specific Glial Tumor Types Are Associated to *BRAF* Mutations

In 2016, the WHO (World Health Organization) classification of central nervous tumors became a more complex histo-molecular classification based on molecular markers specifically paired to histological diagnoses. The more recent WHO 2021 classification now includes low- and high-grade entities strictly linked to MAPK pathway activation, listed in Figure 1 [1,2,16].

Roughly, three types are part of the MAPK pathway activate brain tumors: (1) MAPK-pathway-altered diffuse pediatric LGGs; (2) circumscribed astrocytic gliomas, comprising the PAs, the high-grade astrocytoma with piloïd features (HGAP), and the pleomorphic xanthoastrocytomas (PXA); and (3) indolent epileptogenic lesions in the glioneuronal and neuronal categories with the gangliogliomas (GGLs), dysembryoplastic neuroepithelial tumors (DNET), multinodular and vacuolating neuronal tumors (MVNT), and diffuse leptomeningeal glioneuronal tumors (DLGNT). Among those, we will focus on the *BRAF*-mutant gliomas. 

In the diffuse LGGs, the pediatric-type gliomas are histologically indistinguishable from adult forms, apart from their molecular abnormalities and intra-cerebral locations. They mostly bear a BRAF p.V600E alteration [8,17,18] that might be considered as a specific molecular initiator of gliomagenesis in pediatrics. In fact, adult studies estimate the prevalence of all *BRAF* mutations at less than 1%, whereas in children and adolescents, rates reach 8 to more than 30% of the cases.

In the group of circumscribed astrocytic gliomas, the more frequent alteration is a fusion involving *BRAF* gene and, most frequently, a *KIAA1549* partner, especially in the frequent PAs and in the rare HGAP entity [1,7,19,20]. PXAs, diagnosed in both pediatric and young-adult settings, frequently behave indolently, and are considered as WHO grade 2 tumors. The cases where mitotic activity is higher (5 mitoses per 10 high-power fields) are defined as grade 3 anaplastic gliomas. Most of them carry a BRAF p.V600E mutation combined with a homozygous *CDKN2A* deletion (e.g., 65% of the cases). Extremely rarely, PXA are characterized by a RAF1 or C-RAF fusion [15].

The third group encompassing the spectrum of epileptogenic tumors with the most frequent diagnosis is ganglioglioma (GGL). This brain neoplasm is a WHO grade 1 glioneuronal tumor, typically arising in the temporal lobe of children and young adults and following mostly an indolent course. This tumor has nevertheless the rare possibility of anaplastic transformation in grade 3 cancers. Those grade 1 and 3 forms harbor genetic alterations responsible for MAPK pathway activation, where BRAF p.V600E is evidenced in 10 to 60% cases. Rarely, other SNVs in *BRAF* are described [21,22]. Other epileptogenic indolent tumors are polymorphous low-grade neuroepithelial tumors of the young (PLNTY), DNET, and MVNT, which usually exhibit abnormalities in the MAPK pathway corresponding notably to *BRAF* mutations. 

The high-grade MAPK-pathway-induced gliomas are not considered as a real category in the 2021 WHO classification, but they clearly overlap with the previously described grade 3 PXA or GGL and are mostly enriched in BRAF p.V600E mutated forms [2,22]. The global frequency of this mutation in HGGs is estimated to be 1 to 3% [21,22,23]. A distinct but moving entity is the epithelioid variant of glioblastoma, which highly overlaps with the PXA entity in young adults but presents a better prognosis. In older adults, this variant bears a poor prognosis, as with the IDH-wild-type glioblastomas (GBMs). All epithelioid morphologies seem to be linked with a higher frequency of BRAF p.V600E mutation [24].

## 4. The Concept of Oncogenic-Stress-Induced Senescence Might Be Related to BRAF p.V600E

The oncogenic-stress-induced senescence (OIS) is an antiproliferative response resulting from an altered oncogenic signaling pathway. This OIS is defined as the cellular state where a terminal cell cycle arrest is associated with telomere shortening, limiting neoplastic growth. It is a complex mechanism that is incompletely elucidated and probably promoted by the activation of the RAS/MAPK and PI3K/AKT pathways [25]. In fact, a *BRAF* mutation induces a constant RAS/MAPK activation, leading to a proliferation arrest in cells where p53 and/or p16 are upregulated [25,26]. p16 upregulation inhibits normal CDK4 activity, leading to G1 cell arrest, a cellular state indistinguishable of cellular senescence and it is also responsible for a proteasome-dependent degradation of proteins required for cell cycle progression, mitochondrial functions, cell migration, RNA metabolism and cell signaling [27,28]. This complex mechanism was first described in melanocytic tumorigenesis [27] and was confirmed in gliomas. The concept emerged notably in experiments focusing on neural stem cells where fusion involving *BRAF* gene induced only low-grade glioma-like lesions after engraftments [27,29]. In the same way, it has been shown that transfection of a constitutively active *BRAFv600e* allele into human neurospheres promotes soft agar colony formations, but without a dramatic increase in cell proliferation. After a few culture passages, the *BRAFv600e* transduced cells showed a significant decreased proliferation related to the senescent cell phenotype induced by p16 upregulation [30]. Thus, the initiation and promotion of the low-grade BRAF p.V600E tumors might be limited to a transient increase in proliferation. All those observations led to the concept of OIS in which an activating oncogenic stimulus limits neoplastic growth via the induction of cellular senescence, but this senescent state is not entirely irreversible. Driving glioma progression towards a more aggressive tumor subtype will need additional molecular alterations. In fact, *CDKN2A/B* deletion reverses the senescent state, whereas the loss of p53 does not allow escape from BRAF p.V600E-induced senescence [29]. Nevertheless, the “transformed” cells after OIS might display more aggressive tumorigenic features, a higher capacity of migration, and an increased resistance to anti-tumoral drugs, comparative to cells without initial senescence [31]. Beyond the initial indolent phenotype, OIS is a prerequisite for a more aggressive cell course since the cells surpassed the limited proliferation and it might become a mechanism of therapeutic resistance.

## 5. Prognosis of *BRAF*-Mutant Gliomas

Several studies focusing on those gliomas have pointed out differences in tumor locations and molecular characteristics, as well as their resectability, to explain variabilities in patient outcomes. Therefore, the emerging molecular markers in low- and high-grade gliomas now provide prognostic implications usable routinely in patient therapeutic management. 

In PLGGs, several studies have evidenced that BRAF p.V600E confers poor outcomes when it is compared to *BRAF* wild-type LGGs or those with *BRAF* fusions [32]. More recently, Ryall et al., by studying the clinical outcome of more than 1000 PLGGs, confirmed the poor outcome of *BRAF*-mutant gliomas and proposed a molecular-based risk stratification pushing forward BRAF p.V600E gliomas into the intermediate- and high-risk subgroups [8]. The prognostic impact of those *BRAF*-mutant PLGGs has been modified since the tumor was entirely resected and then reached the prognosis of *BRAF*-fused tumors. In fact, the other MAPK-altered LGGs are mostly distributed in the low- or intermediate-risk subgroups. This prognosis will be also modulated by the associated molecular abnormalities. Thus, *CDKN2A* deletion is the main other molecular alteration described among PLGGs and accentuates the worse prognosis in BRAF p.V600E tumors [22,32,33]. It sustains the fact that *CDKN2A* deletion is usually associated with anaplastic transformation in BRAF p.V600E PLGGs [22]. 

Regarding MAPK-altered high-grade gliomas and compared to histone-mutated tumors, BRAF p.V600E mutation drives a better prognosis connecting those malignant forms to specific high-grade genotypes and immune microenvironments [34]. When BRAF p.V600E is present in tumor cells, as already described above, it usually promotes a modest proliferative cell capacity, probably explaining the better prognosis of patients, who experience an improved survival outcome with an indolent tumor course [35]. Furthermore, rare preclinical studies proposed a higher sensitivity of those tumor cells to radiation and an increased radiosensitization in the case of concomitant BRAF inhibition [36,37,38]. 

## 6. Additional Molecular or Passenger Alterations Lead to the Disruption of the Balance between Cell Senescence and Proliferation in BRAF p.V600E Glioma Mutants

The slow-growth tumor phenotype induced by the initiation of the BRAF p.V600E mutation seems to be partially reversible due to the additional molecular abnormalities that guide a more aggressive evolution. Those co-alterations cover, mostly, the NF1 and mTor pathways, but also frequently exhibit the homozygous loss of *CDKN2A*, amplification of CDK4, TERT upregulation or ATRX downregulation, epigenetic modulation, and DNA repair abnormalities [39]. All those abnormalities are listed in Table 1.

### 6.1. CDKN2A Deletion and Its Impact on CDK4/6 Function

p16, encoded by the *CDKN2A* gene, is a major tumor suppressor protein, highly expressed in senescent cells in in vitro models and inactivated in a variety of human cancers, including 30 to 70% of pediatric or adult high-grade astrocytomas [21,33,40]. The loss of p16, mostly related to a *CDKN2A* deletion, abrogates the senescent features of tumor cells stably overexpressing altered BRAF. In fact, the study of Raabe et al. on 66 PAs evidenced that the loss of expression is present in 14% of cases and the outcome analyses confirmed that 33% of patients losing p16 expression died from their disease, compared to the 3.6% of deceased patients still expressing p16 in their tumor [30]. This downregulation is also frequently present in BRAF p.V600E gliomas, such as anaplastic forms or in pediatric diffuse MAPK-altered gliomas, GGLs and PXAs [19]. Furthermore, in the case of the rare aggressive progression in PLGGs (26 anaplastic transformations out of a large cohort of 886 PLGGs with long-term clinical follow-up) [22], the most frequent genetic co-occurrences were the association of a BRAF p.V600E mutation and *CDKN2A* deletion. It is important to note that *CDKN2A* deletion can be acquired already at the low-grade step before becoming an aggressive cancer, and then, being used as a supplementary and independent prognostic biomarker in those clinical situations [22]. It clearly worsens the poor outcome of *BRAF*-mutant PLGGs [40,41]. 

In HGGs with piloïd or epithelioid features, more than 60% of them present a deletion of *CDKN2A*, which co-occurs in half of them with the activation of the MAPK pathway [19,40]. The *CDKN2A/B* homozygous deletion is also identified independently from BRAF activation as a biomarker of high-grade astrocytomas with a worse clinical outcome. Usually, the loss of this gene in those HGG entities is considered in parallel to other independent factors such as the concurrent gain of whole chromosome 7, the loss of whole chromosome 10, including the *PTEN* gene, TERT promoter mutations, and/or EGFR amplification [39,40,41,42]. Despite the co-occurrence of *CDKN2A* loss and BRAF mutations, patients’ outcome do not worsen as the tumors are still predominantly driven by BRAF mutation and, subsequently, evolve with a more indolent course [34,40]. 

Further to the *CDKN2A* gene status, only a little data on PLGG or HGG focus on the p16 protein expression that might be made extinct by other molecular mechanisms (e.g., gene promotor silencing, post-transcriptional abnormalities or secondary to their regulators) [43,44]. 

Nevertheless, as *CDKN2A* loss shapes glioma cells into a higher-grade phenotype, it subsequently interacts with CDK4/6 expression in those tumors. Thus, CDK4/6 overexpression/amplification might co-occur during *BRAF*-mutant gliomagenesis and increase their aggressiveness. CDKN2A inhibits the expression of CDK4/6 during the cell cycle steps. Therefore, CDK4/6 activation should be frequently described, but, for now, only a few publications observe their interplays in those HGG entities [45,46]. CDK4 overexpression seems to be an enhancer of in vitro glioma colony formation, pushing glioma cell proliferation and extending their resistance to the temozolomide commonly used in HGGs [45]. The CDK4 knockdown might impede its role in an MAPK-activated HGG and be a way to surpass therapeutic resistance to standard therapies. 

The other proliferative factors, regularly deregulated in adult and pediatric HGGs (e.g., MYC, MDM4, CDK1), were rarely studied in BRAF p.V600E mutated gliomas, but their paired genes can exhibit high level of amplifications [33,34].

Beyond *CDKN2A* loss and *CDK4/6* amplification, additional abnormalities such as *ATRX* loss or *TERT* promoter mutations might co-occur and balance those gene copy number variations (CNVs). 

### 6.2. Telomerase Reverse Transcriptase (TERT) Activation and ATP-Dependent Helicase (ATRX) Mutations Are Mutually Exclusive

Telomerase reverse transcriptase (TERT) plays a key role physiologically in maintaining the telomerase length of the chromosomes. Initially found in melanoma and thyroid cancers, recurrent *TERT*-activating promotor mutations were then described in numerous cancers, including adult GBMs and anaplastic oligodendrogliomas. Interestingly, in melanoma and thyroid papillary carcinoma, *TERT* mutation is widely found to be associated with BRAF p.V600E mutations [33,47,48]. This co-occurrence is usually observed at the metastatic stages and significantly linked to an adverse prognosis. Therefore, *BRAF* and *TERT* promoter mutations appeared as a fundamental genetic background that cooperatively drives progression and aggressiveness, even when *CDKN2A* loss is already present. These simultaneous abnormalities were reported in small numbers of PXAs, anaplastic PXAs (aPXAs), and epithelioid GBMs. *TERT* alterations are identified across all age groups [15,48]. Nevertheless, *TERT* amplifications were described and specifically present in very young patients, whereas its promoter mutation was identified much more in adolescents and young adults [34,49,50]. It can be associated with almost 50% of the *BRAF*-mutant HGGs. Recent molecular insights in aPXAs demonstrated the links between these two mechanisms. The mutated BRAF protein phosphorylates and activates FOS, which acts as a transcription factor and binds the GABPB promoter to increase its expression. Thus, it drives the formation of the GABPA/GABPB complex, which selectively binds and activates the mutant *TERT* promoter and upregulates TERT expression [51]. This overexpression leads to the suppression of cell apoptosis, which subsequently involves the direct transcriptional regulation of survivin and TRAIL-R2 by TERT activation [52]. This alteration of *TERT* in *BRAF*-mutant HGGs suggests the importance of telomere maintenance in additional to an activated proliferation provoked by *CDKN2A* loss in gliomagenesis. 

To maintain the length of telomere, the alternative lengthening of telomere (ALT) mechanism is another molecular process where telomeric DNA on one chromosomal arm is used as a template for the DNA-polymerase-mediated TERT-independent extension of shortened telomeres on a different chromosomal arm. The so-called ALT phenotype was uniformly associated with loss-of-function mutations in *ATRX* which are considered as a common surrogate marker of this phenotype [53]. Those alterations were mostly observed in histone *H3*- or *IDH1*-mutant HGGs. Nevertheless, few publications identified *ATRX* mutations co-occurring with BRAF p.V600E in rare tumors without *TERT* promoter mutations [54]. 

### 6.3. BRAF/ERK and Pi3K/AKT/mTOR Pathways Cooperate in the Tumorigenesis of Gliomas

mTOR is a serine/threonine kinase that functions as a central regulator of cell-growth-related processes (proliferation, stemness features, metabolic cell adaptation and switch, interactions with microenvironment). In physiological conditions, BRAF/ERK and mTOR pathways are not strictly separated and several mechanisms of cross-inhibition, cross-activation, or pathway convergence on substrates are now elucidated [42,55]. In gliomas, the activation of the mTOR pathway, mostly demonstrated in the immunohistochemical overexpression of various effectors, is highly present, but, for now, no extensive data link the Pi3K/AKT/mTor deregulation signature to a specific MAPK-pathway driver in high-grade and low-grade gliomas [56]. The studies on PLGGs have then highlighted a global and frequent overexpression of various mTOR effectors such as pS6 or RICTOR, demonstrating that both mTORC1 and mTORC2 complexes are involved in fused PAs or in the context of neurofibromatosis type 1 (NF1) abnormalities [56]. In GGLs and DNETs, the presence of a *BRAFv600e* mutation was significantly associated with the increased expression of phosphorylated ribosomal S6 protein, and together, they worsen the post-operative seizure outcome of patients [57]. In a preclinical setting, mutated *BRAFv600e* expression acts in concert with AKT/mTor signaling to elicit benign tumors in murine models, comprising dysmorphic neurons and astroglial cells recapitulating GGLs [58]. All those sparse observations are in favor of the early involvement of the AKT/mTor pathway in *BRAF*-mutant glioma development. Another central factor of this pathway is that PTEN is rarely associated with BRAF-mutant LGGs, except in the case of the worst outcomes [39].

On the contrary, in pediatric or adult HGG, a modulation of the *PTEN* gene was mostly observed in association with mTOR pathway activation, leading to the frequent use of this target [59]. This additional activation of the Pi3K/AKT/mTOR signaling pathway seems to exacerbate the cooperation with BRAF activation and the promotion of tumor cell proliferation. Consistent with melanoma publications, most of the glioma studies postulated that the decrease activity of PTEN is related to tumor progression and that the activation of the mTOR pathway was able to override *BRAFv600e*-induced senescence [15,33,60]. This mTOR activation might also give mutant cells a propensity to adapt cell metabolism, which leads to therapeutic resistance [61]. 

### 6.4. NF1 Status in BRAF p.V600E Glioma Mutants

As an important part of RAS signaling activation, *NF1* can be deleted or mutated somatically or at the constitutional level in gliomas. When considering its associated molecular deregulation with *BRAF* mutations, the global literature on cancers differentiates the frequent class I *BRAFv600* mutants and class II mutations from class III *BRAF* mutations because of their independence from NF1/RAS deregulation [62]. The *BRAFv600* tumors bear a high kinase activity that does not need NF1 abnormalities, supporting the fact that *BRAF* mutants are usually rare in the spectrum of NF1 disease [8]. NF1/RAS deregulation in glioma class III mutations might be, as in melanoma or lung cancers, a way to resist to molecules inhibiting BRAF, but no recent publications link specific alterations to their therapeutic responses. 

### 6.5. Epigenetic and Hypermutator Phenotypes in BRAF p.V600E Glioma Mutants

Almost no specific epigenetic nor hypermutator phenotype was described in the literature on *BRAF*-mutant PLGGs, whereas few data are now available on those components notably impacting immune modulation in HGGs. Only one work focused on PAs, which presented a specific hypomethylation signature independently from tumor location. The loss of trimethylation in pediatric HGGs is a key diagnostic factor used by pathologists and linked to PRC2 and EZH2 modulation [63]. Thus, EZH2 expression levels in gliomas increase within tumor grades. Its overexpression occurs in all pediatric HGGs and in adult GBMs, especially those bearing epithelioid features (present in 50% of the cases) [64]. It is observed in 20% of PXAs, co-existing frequently with BRAF p.V600E mutations. Therefore, strong EZH2 expression, a high Ki-67 index and BRAF p.V600E mutations were significantly associated with a decreased overall survival. 

Along with those epigenetic variations, the other characteristic in a subset of HGGs is the presence of a high mutational burden [65], which is defined by an elevated neoantigen load and a pronounced immune response, called hypermutator HGGs. In the MacKay et al. publication in 2018, four pediatric *BRAF*-mutant cases were classed as hypermutators and were significantly associated with a high percentage of CD8 positive cells in the central area of the tumor. Thus, PXA-like tumors characterized by this *BRAF* mutation might be named as immune warm gliomas, which might partially explain their better response to therapies associating radiotherapy and temozolomide. 

In the same way, data on adult GBMs exploring immune pathways showed that *BRAF*-mutated tumors, compared to EGFR and IDH1 mutants, were particularly highly overexpressed for immune functions such as T cell modulation or interleukin signaling [24]. No hypermutator phenotype, nor hypomethylation data are available for now, nor DNA repair mechanisms involved specifically in those MAPK-activated adult gliomas.

All this molecular knowledge is now making neuro-oncologists think about new targeted options for those MAPK-activated gliomas and to go further using therapies in combination to surpass secondary resistances. 

## 7. How to Treat Those MAPK-Activated Gliomas with *BRAF* Mutations?

### 7.1. Conventional Therapies

In unresected PLGGs bearing BRAF p.V600E, chemotherapeutic protocols were, in the past, the standard first-line approach. The chemotherapy strategies showed variable levels of effectiveness in clinical tumor courses ranging from prolonged growth arrest to continuous progression or multiple recurrences. The types of chemotherapies were not the key, as a specific drug combination or regimen did not affect PFS and OS in those patients. Among the past and more recent large studies, independently from the chemotherapies, the 5-year progression free survival (PFS) rates have remained stable at around 30 to 40%. Furthermore, those treatments have short- and long-term effects that conduct with such weak PFS as to promote the use of alternative targeted therapies, to improve tumor responses and find a prolonged and definite cure. In adult LGGs, as in pediatric forms, the first step tends to an extended resection and discussion of innovative strategies [8,32,66,67]. 

For adult HGGs or GBMs, the gold standard in first-line treatment has remained an association with chemotherapy (e.g., temozolomide), and radiotherapy based on the Stupp scheme since 2005 [68]. In pediatric sus tentorial HGG, an adapted Stupp schedule with 12 courses post-radiotherapy was also set up as a standard [34]. Nevertheless, as this therapeutic strategy was not curing patients and with the era of BRAF inhibitors, the concept of targeted therapies also emerged and is now a validated option [66,67]. 

### 7.2. Therapies Targeting BRAF Altered Gliomas

Mostly based on the experience of melanoma treatment and the recent clinical trials in adult [66,67] and pediatric (NCT02684058) settings, targeted therapies will rely both on the exact nature of the activating BRAF alteration and on the possible additional alterations. 

The co-alterations to BRAF mutations may be present at diagnosis or may occur during the progression of the disease, eventually under targeted therapy pressure and be responsible for acquired resistance. The alteration leading to the activation of the MAPK pathway does not activate the downstream effectors via the same mechanism and consequently the targeted therapies might be driven by those differences. Notably, regarding *BRAF* mutations, various activating alterations are currently grouped into three classes, based on their dependence on dimerization and on activation by upstream RAS for their activity [66,67]. The *BRAF* class I mutation corresponds to the more frequent SNV that is V600E. This variant activates downstream MEK, as a monomer, independently of the upstream RAS activity. The class II alteration is mostly linked to an in-frame deletion in BRAF and most fusions (e.g., the most frequent is KIAA1549-BRAF fusion), but also rarer non *BRAFv600* mutations. Thus, the BRAF regulatory domain is lost, leading to an increase in the affinity for its dimerization and allowing a higher BRAF kinase activity independently from upstream RAS activation. The class III mutations lead to the impairment of its kinase activity, but the *BRAF*-mutant binds then even more tightly when dimerized and increases the activation of the wild-type binding partner (B-RAF, A-RAF or C-RAF). The class III mutations often arise in conjunction with other alterations that increase upstream RAS activity, such as a tyrosine kinase receptor (TKR) mutation or amplification, NF1 loss, or an RAS mutation itself.

Currently, two kinds of MAPK inhibitors are available in routine practice in various cancers: one is targeting RAF and the other MEK. The BRAF inhibitors (BRAFi) are ATP-competitive small molecules that selectively bind to and inhibit RAF monomers. BRAFi are only effective in cells where ERK is activated by the BRAF p.V600E mutation itself. In the case of wild-type BRAF or non BRAF p.V600E mutation, BRAFi lead to a paradoxical increase in ERK signaling by facilitating the formation of RAF dimers, especially B-RAF-C-RAF, able to accelerate tumor growth in vivo [62]. For this reason, BRAFi, such as the FDA-approved molecules vemurafenib, dabrafenib or encorafenib, should only be used in tumors with this BRAF p.V600E mutation. The second compounds target MEK and correspond to allosteric inhibitors preventing the conformational change of MEK into its active form. Those MEK inhibitors (MEKi, such as trametinib, binimetinib, selumetinib, cobimetinib) are potentially effective against tumors harboring mutations upstream of MEK, including RAS mutation, type I and type II BRAF alterations, TKR alterations, or in the case of NF1 loss. The third generation of pan-RAF inhibitors are also starting to show attractive results in clinical studies in both mutant and fused gliomas [69]. Recently, various new small molecules harboring other mechanisms, such as “paradox breakers” or “dimer disrupters”, have been evaluated which can interrupt B-RAF dimerization through the disruption of the αC-helix or inhibition of both wild-type RAF dimer partners and the monomeric active class I mutant BRAF, respectively [70,71]. 

Those compounds have since fueled multiple trials in various cancers, including LGG and HGGs, bearing those mutations. In fact, our first medical successes with those MAPK-pathway-targeted therapies were evidenced in melanomas bearing BRAF mutations, where BRAFi demonstrated early responses in monotherapy. Nevertheless, we also saw melanoma resistances emerging in most patients after an average of 6-month treatment, increasing the use of up-front BRAFi and MEKi combinations. This strategy delayed the occurrence of therapeutic resistances and improved PFS and overall survival (OS) in patients with advanced melanoma [72]. Based on this knowledge, the use of those compounds was expanded to diseases bearing the same mutations. Thus, several case reports or small series have described encouraging responses in relapsing BRAF p.V600E HGGs, particularly using the double BRAF/MEK blockade therapy. It allowed a prolong disappearance or stabilization of the brain tumors in adult and pediatric patients [22,72,73,74,75]. Nobre et al. [75] collected clinical, imaging, molecular and outcome information from pediatric patients with low- and high-grade BRAF p.V600E-mutated gliomas treated with a BRAF inhibitor (dabrafenib or vemurafenib). Most PLGGs showed sustained responses to the BRAFi, independently from the histological subtypes, with an objective response observed in 80% of the cases and a complete response in half of the studied population. Concomitant *CDKN2A* deletion did not appear as a predictive molecular marker of tumor resistance or unresponsiveness. In pediatric HGGs, responses were also observed but were only transient and in a lower percentage of patients (e.g., response rate of 36%). All children finally experienced re-progression and died of their disease. Therefore, tumor grades appeared as a stronger predictor of response to targeted monotherapy in the pediatric cohorts. In addition, the encouraging results in PLGGs must be tempered by the frequent secondary re-progression experienced after discontinuation of the monotherapy. Nevertheless, in the end, those PLGG re-progressions responded if rechallenged with BRAFi alone or in combination with MEKi. All those pooled results worked to promote first-line bitherapies as in the international study NCT02684058 [76]. The combination is becoming a standard with the upcoming marketed authorization of dabrafenib plus trametinib in first-line mutant LGGs and as a second-line therapy in HGGs. The era of pan-RAF kinase inhibitors also allows new strategies and will probably improve the tolerance while lowering resistances [61,73,77]. Those resistances mainly originate in the MAPK pathways when NF1 or PTEN expression is lost or through the acquisition of secondary mutations in BRAF mutated genes.

Thus, the extended use of such new targeted approaches will afford new insights and progressively decipher the adequate recommendations as to how we should administer those new compounds and determine the co-alteration signature of sensitivity. 

## 8. Conclusions

Collectively, our review reveals the specific role of BRAF mutations as a major molecular driver in PLGG and HGG gliomagenesis, impacting the parallel progression and prognosis of those brain tumors. The BRAF p.V600E mutation is frequently associated with passenger molecular abnormalities that lead to an aggressive and/or resistant cell phenotype to current therapies. Those associated alterations cover multiple pathways from cell cycle to epigenetic effectors, as well as mTOR pathways or mechanisms involving TERT and ATRT functions. Clearly, this recent molecular knowledge opens the path for targeted therapies upfront in those gliomas, taking into account driver but also passenger mutations or deregulations for a precision medicine adapted to each patient.

## Figures and Tables

**Table 1:** Summary of all co-alterations evidenced in BRAFv600-mutant gliomas and their impact.

	Alterations	LGG vs. HGG	Cell Phenotype	Outcome	Therapeutic Resistance
ARAF/CRAF	amplification	HGG	no data	Worst	no data
CDKN2A/B	deletion	LGG HGG	progression progression	Worst	no data
CDK4/6	overexpression	HGG	progression	no data	resistance to temozolomide
TERT	mutation or amplification	HGG	progression	no data	no data
ATRX	mutation	HGG	progression	no data	no data
mTor	activation	LGG HGG	progression	no data	no data
PTEN	deletion	LGG HGG	progression progression	Worst no data	resistance to chemotherapy no data
NF1	deletion	LGG HGG		no data no data	resistance to targeted drugs in class III mutation
EZH2	overexpression	HGG	progression	Worst	no data
immune CD8 cells	presence	HGG	no data	better	higher response to radiotherapy and chemotherapy

## Data Availability

The data presented in this study is available within the article.
